# Structure of the protective nematode protease complex H-gal-GP and its conservation across roundworm parasites

**DOI:** 10.1371/journal.ppat.1008465

**Published:** 2020-04-09

**Authors:** Charlotte A. Scarff, Rebecca F. Thompson, George F. J. Newlands, Alexander. H. Jamson, Christopher Kennaway, Vivian J. da Silva, Elida M. Rabelo, Chun-Feng Song, John Trinick, W. David Smith, Stephen P. Muench

**Affiliations:** 1 School of Molecular and Cellular Biology, Faculty of Biological Sciences & Astbury Centre for Structural Molecular Biology, University of Leeds, Leeds, United Kingdom; 2 Moredun Research Institute, Midlothian, Scotland; 3 School of Biomedical Science, Faculty of Biological Sciences & Astbury Centre for Structural Molecular Biology, University of Leeds, Leeds, United Kingdom; 4 Departamento de Parasitologia, Instituto de Ciências Biológicas, Universidade Federal de Minas Gerais, Belo Horizonte, Minas Gerais, Brazil; 5 Laboratory *Center* of *Electron Microscopy*, *Hebei Medical University*, Shijiazhuang, P.R.China; University of California, San Francisco, UNITED STATES

## Abstract

Roundworm parasite infections are a major cause of human and livestock disease worldwide and a threat to global food security. Disease control currently relies on anthelmintic drugs to which roundworms are becoming increasingly resistant. An alternative approach is control by vaccination and ‘hidden antigens’, components of the worm gut not encountered by the infected host, have been exploited to produce Barbervax, the first commercial vaccine for a gut dwelling nematode of any host. Here we present the structure of H-gal-GP, a hidden antigen from *Haemonchus contortus*, the Barber’s Pole worm, and a major component of Barbervax. We demonstrate its novel architecture, subunit composition and topology, flexibility and heterogeneity using cryo-electron microscopy, mass spectrometry, and modelling. Importantly, we demonstrate that complexes with the same architecture are present in other Strongylid roundworm parasites including human hookworm. This suggests a common ancestry and the potential for development of a unified hidden antigen vaccine.

## Author summary

Roundworm parasites are responsible for disease within both animal and human populations with Ancylostomiasis (human hookworm) affecting over 500 million a year and the Trichostrongylids causing significant animal health and production harm to global livestock industries. Despite their prevalence many aspects of roundworm parasite growth and homeostasis are poorly understood. For example, we still do not understand how these parasites digest their meal (often blood) and take up nutrients such that they avoid immune system recognition by not releasing the degradative enzymes into the blood stream. In this study we have, for the first time, solved the structure of the *Haemonchus* galactose containing glycoprotein complex (H-gal-GP) and show how it acts as an efficient digestion machine, capable of trapping hemoglobin and channeling it to different enzymes for processing. Moreover, we show for the first time that this is conserved across other important roundworm parasites (taxonomic Order Strongylida), suggesting a common digestive mechanism. Importantly, H-gal-GP is an active ingredient in the Barbervax vaccine and the conservation of this complex across different parasites could open up new avenues for developing a universal vaccine against these devastating roundworm parasites.

## Introduction

Roundworm parasites, particularly gastrointestinal species, are the most important cause of livestock disease affecting the world’s poor, causing greatly reduced production efficiency [1, 2] and as such are a threat to global food security. They are also important causes of veterinary disease in high income countries, costing the Australian sheep industry >$430 million per annum [3, 4]. Ancylostomiasis caused by hookworms is one of the most prevalent human parasitic diseases in the world and causes anemia and malnutrition among the poorest populations. The disease affects over 500 million people in tropical and subtropical regions of the world [5] and 5 billion people are at risk of infection worldwide [6]. In the past, roundworm parasites have been largely controlled by broad spectrum anthelmintic drugs but resistance to these is now common [7–9]. Alternative methods of control are urgently needed. One approach is vaccination, where the use of hidden antigens, components of the worm gut not encountered directly by the host’s immune system, has been explored. To date only two vaccines are available commercially for any roundworm parasites of any host (Bovilis Huskvac and Barbervax, for sheep and goats) and a vaccine based on hookworm gut-expressed antigens is in Phase I clinical trials [10].

Barbervax is a hidden antigen vaccine that is specific for *Haemonchus contortus*, commonly known as the Barber’s Pole worm, a hematophagous nematode that parasitizes the abomasum (true stomach) of sheep and goats. *H*. *contortus* is the nematode ‘‘nemesis” of small ruminant production systems in tropical and subtropical regions of the world [11]. Barbervax contains a mixture of integral membrane glycoprotein antigens isolated from adult *Haemonchus* intestinal cells, formulated with saponin as adjuvant. A major Barbervax component is H-gal-GP (*Haemonchus* galactose containing glycoprotein complex), which is highly protective in sheep vaccine trials [12–14]. H-gal-GP is located in the luminal brush border of the nematode gut and digests ovine hemoglobin and albumin, major components of the *Haemonchus* blood meal [12–15]. A combination of SDS-PAGE, N-terminal sequencing and cDNA library screening, have shown that H-gal-GP is composed primarily of families of two aspartyl proteases (PEPs 1 or 2), four metalloproteases (MEPs 1, 2, 3 or 4) and several cysteine proteinases (CPs) [16–18]. As a denatured immunogen, H-gal-GP is far less protective suggesting conformational epitopes are important, or that the crucial protective antigenic site is at an interface between two or more subunits [17, 18]. Attempts at protection with recombinant forms of the component enzymes have been unsuccessful, even when delivered in combination [17, 19, 20]. Barbervax contains the native antigen purified from adult parasites.

H-gal-GP is closely related to another *Haemonchus* complex that is also a highly protective antigen, H-sialgal-GP [21]. H-gal-GP is purified by binding to peanut lectin, which preferentially binds galactosyl (β-1,3) N-acetylgalactosamine, whilst H-sialgal-GP binds to jacalin lectin that preferentially binds sialylated versions of this glycan. H-sialgal-GP differs from H-gal-GP in that it lacks the 230 kDa component, shown by N-terminal sequencing to contain MEPs 1, 2 and 4 [21]. However, MEPs 1, 2 and 3 were identified in H-sialgal-GP by LC-MS-MS (S2 Table). Sequence data suggest that other important parasitic nematodes are likely to contain similar complexes to H-gal-GP and H-sialgal-GP, including *Ancylostoma ceylanicum* which infects both dogs and man, *Teladorsagia circumcincta*, which infects sheep and goats, *Ostertagia ostertagi*, which infects cattle, and *Ascaris suum*, which infects pigs [18].

Given the importance of H-gal-GP in the hidden antigen vaccine Barbervax, we sought to determine the quaternary structure of the complex to inform on function. Here, we reveal the structure of H-gal-GP by cryo-electron microscopy (cryo-EM) and use negative-stain EM to show that *T*. *circumcincta*, *O*. *ostertagi* and *A*. *ceylanicum* all have complexes with a similar overall architecture. The quaternary structure of H-gal-GP revealed here suggests it functions as an efficient degradative machine and shows why recombinant constituents are not an effective vaccine. Its effectiveness as an immunogen and conservation across species holds out promise of new vaccines and inhibitors against roundworms of human and veterinary importance.

## Results

### Cryo-EM structure of H-gal-GP

**Fig 1:**
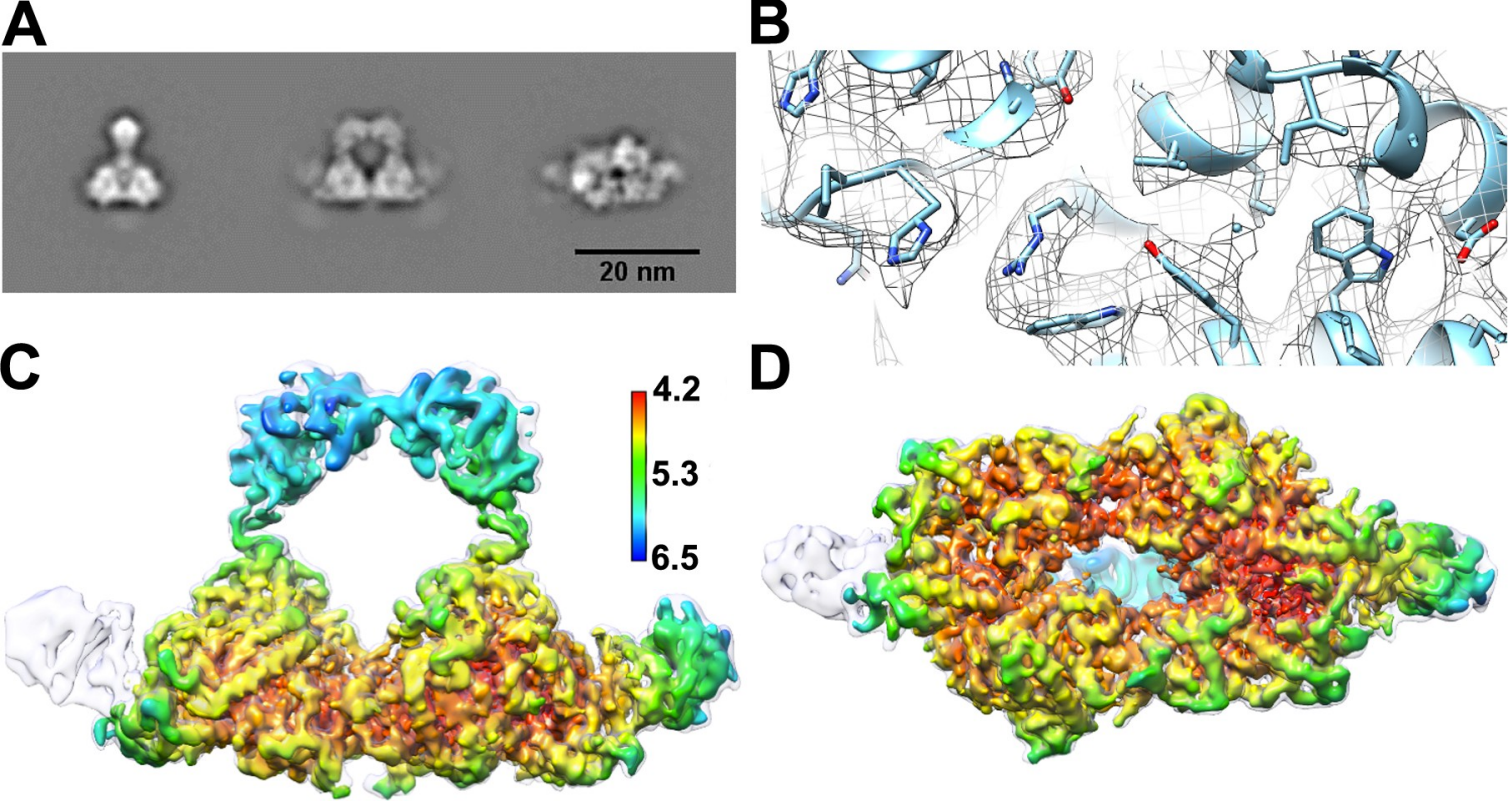
Cryo-EM structure of H-gal-GP at 4.5 Å global resolution. **A** Representative 2D class averages of two side and an end view. **B** Representative region of electron density showing the presence of density for the bulkier amino acids. EM density for H-gal-GP colored by local resolution as seen from the side **C** and end **D**, overlaid with EM density from second map at 6.2 Å global resolution (light grey) containing second wing of density.

H-gal-GP complex was extracted and purified from detergent extracts of *Haemonchus contortus* membranes by peanut lectin affinity chromatography as previously described [21]. Cryo-EM grids were prepared as described in the methods with particles showing a good distribution within the ice and a low level of aggregation (S1A Fig). Visual inspection of the raw micrographs of H-gal-GP showed three distinct appearances, two side views and an end view with the resulting classes showing that these views represented 15%, 30% and 31% of the particles respectively (Fig 1A & S1B Fig). The 2D averages were well defined and showed clear secondary structure features. 3D classification and refinement yielded an EM density map at a global resolution of 4.5 Å with higher resolution within the core of the complex (Fig 1, S1B Fig, S1 Movie and S1 Table).

Upon 3D classification it was apparent that significant heterogeneity was present within the dataset. The final reconstruction of H-gal-GP at 4.5 Å global resolution (Fig 1) was extracted from approximately 60% of the particles by classification with masking of the core base region. Without masking, a second reconstruction at 6.2 Å global resolution containing extra density was obtained (30% of particle dataset; Fig 1 and S1C Fig). The reconstruction revealed that H-gal-GP has, to the best of our knowledge, a novel architecture, a central base with two ‘wings’ and an archway, giving rise to a cavity in the middle of the complex. The reconstruction is ~260 Å long, ~110 Å wide and ~150 Å high, with the cavity being approximately 60 Å^3^. The base has a series of channels connecting to the central cavity. The two wings of density, although similar, have slight differences in volume and shape and are of significantly lower resolution than the core of the complex. In the 2D image averages, the wings are less well defined than the base or archway suggesting they are flexible or compositionally heterogeneous, in agreement with the outcome of 3D classification. Poorly resolved density can also be seen protruding from the base in the 2D class averages that is not resolved in the 3D reconstruction. This density is explored further below.

Processing of data obtained from a separate data collection for the related H-sialgal-GP produced a near identical 3D reconstruction, to ~7 Å resolution (see S1D & S1E Fig). Thus, the quaternary structure of H-sialgal-GP and H-gal-GP are very similar, consistent with the sequence conservation between the complexes.

### Subunit identification, modelling and fitting

**Fig 2:**
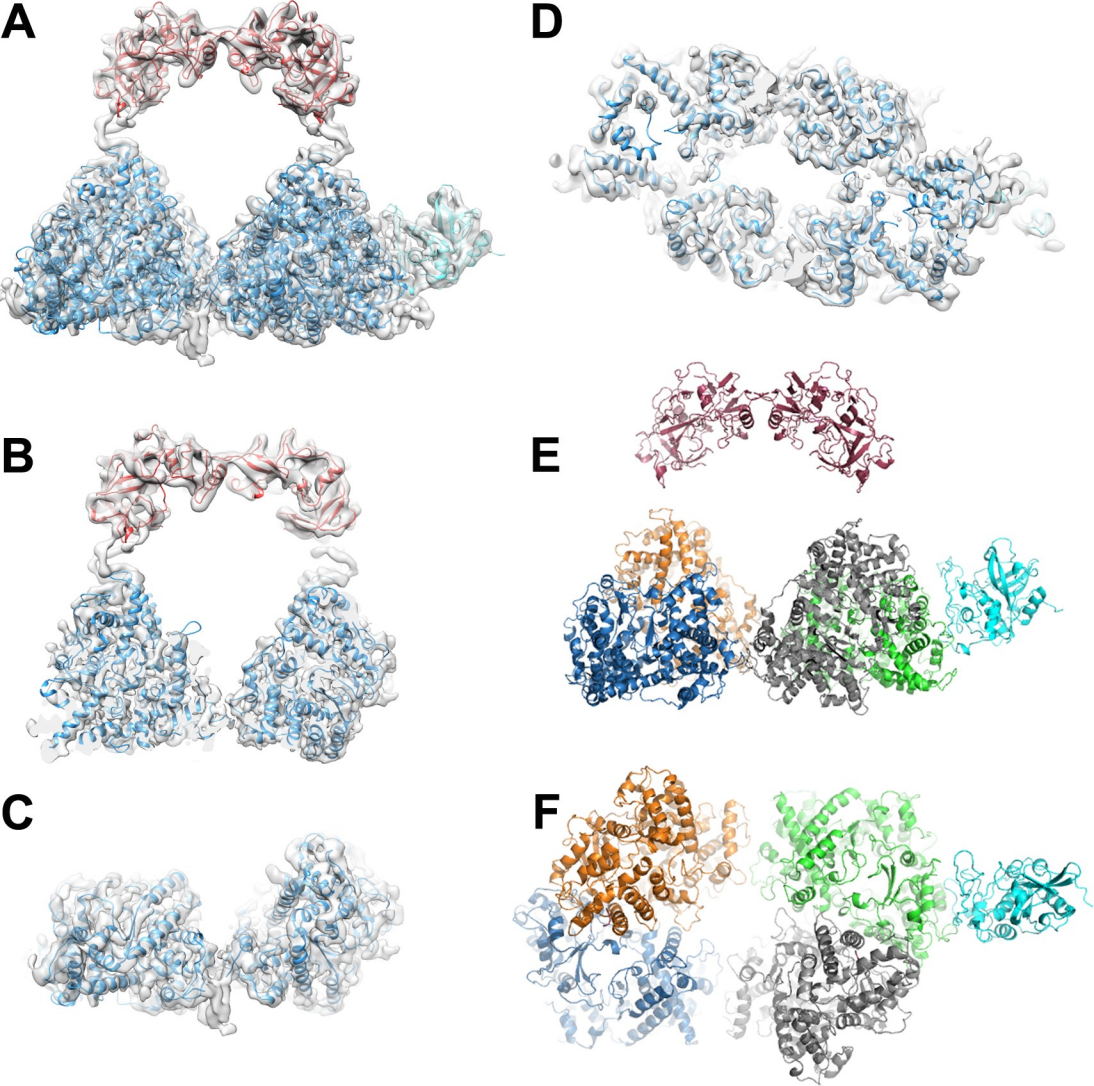
Subunit fitting to H-gal-GP EM density. **A** EM density of one-winged H-gal-GP (4.5 Å map) fitted with models of aspartyl protease PEP 1 (red), MEP3 (dark blue) and cysteine protease (cyan). A cropped view of **A**, through the centre of the H-gal-GP complex (**B**) and cropped to just show two of the MEP3 domains (**C**). **D** H-gal-GP map viewed from the base showing the fitting of the MEP subunits. Fitted subunits of the H-gal-GP complex viewed from the side (**E**) and base (**F**) and colored red (PEP1), cyan (cysteine protease) and orange, blue, green and grey for the four MEPs.

Previous analysis of the components of H-gal-GP has been conducted by use of SDS-PAGE and N-terminal sequencing. To confirm the identity of the subunits present in H-gal-GP and H-sialgal-GP purifications, they were analysed by SDS-PAGE followed by gel band extraction, tryptic digest and liquid chromatography-tandem mass spectrometry analysis where possible (S2 Fig and S2 Table). All MEPs, except MEP4, were identified in H-sialgal-GP preparations, as well as PEP1 and PEP2 and the cysteine protease, Peptidase C1A domain containing protein (Genbank:CDJ88569.1) with MEP4 identified in H-gal-GP only [17]. No tertiary structure information is available for any of the H-gal-GP subunits, however BLAST searches [22] revealed they displayed significant sequence similarity to previously solved crystal structures from other species. Therefore, modelling was performed with Phyre2, a protein modelling web server [23], which made models for MEP1, MEP2, MEP3, MEP4, parasite pepsinogen (PEP1), aspartyl protease (PEP2) and the CP peptidase C1A domain containing protein. The resulting models showed good primary sequence coverage and high confidence values (S3 Table). The models for each of the MEPs were all very similar (S2 Fig). The sequence conservation between the MEPs is approximately 60% and multiple sequence alignment shows they have highly conserved regions [18]. The only striking difference between the MEPs is that MEP4 has an N-terminal insert of approximately 100 residues, which could not be modelled (S3 Table). The resulting homology models were docked into the reconstruction manually with rigid body refinement subsequently conducted in UCSF Chimera [24]. Once docked, models were refined with molecular dynamics flexible fitting (MDFF) [25]. Both aspartyl proteases (PEP1 and PEP2) fit well within the arch, which is not sufficiently bulky to accommodate any of the metalloproteases (Fig 2). The PEP1 model was docked into the map as the model for PEP2 was almost identical. Since the MEP models were all similar in tertiary structure, four copies of MEP3 were docked into the central base. Fitting of MEP3 to the core of H-gal-GP showed very good agreement with the determined map (Fig 2). A single CP was accommodated with reasonable agreement into the wing. EM density between that filled by the PEP1 model and MEP3 models likely accommodates a cysteine-rich region in PEP1 that is not included in the PEP1 model (residues 107–143, S3 Table).

H-gal-GP was previously estimated to be approximately 1 MDa in mass from calibration of native blue gels [16]. H-gal-GP is known to be extensively glycosylated but the mass of glycans estimated from previous studies [16, 18] and the masses of subunits docked into the EM density suggest that H-gal-GP is only approximately 0.5 MDa in mass (see S4 Table). The discrepancy in mass calculation from native gels is most likely due to the large central cavity making the complex run slower on gels than a more compact structure of the same mass.

### Functionality and localisation of H-gal-GP

**Fig 3:**
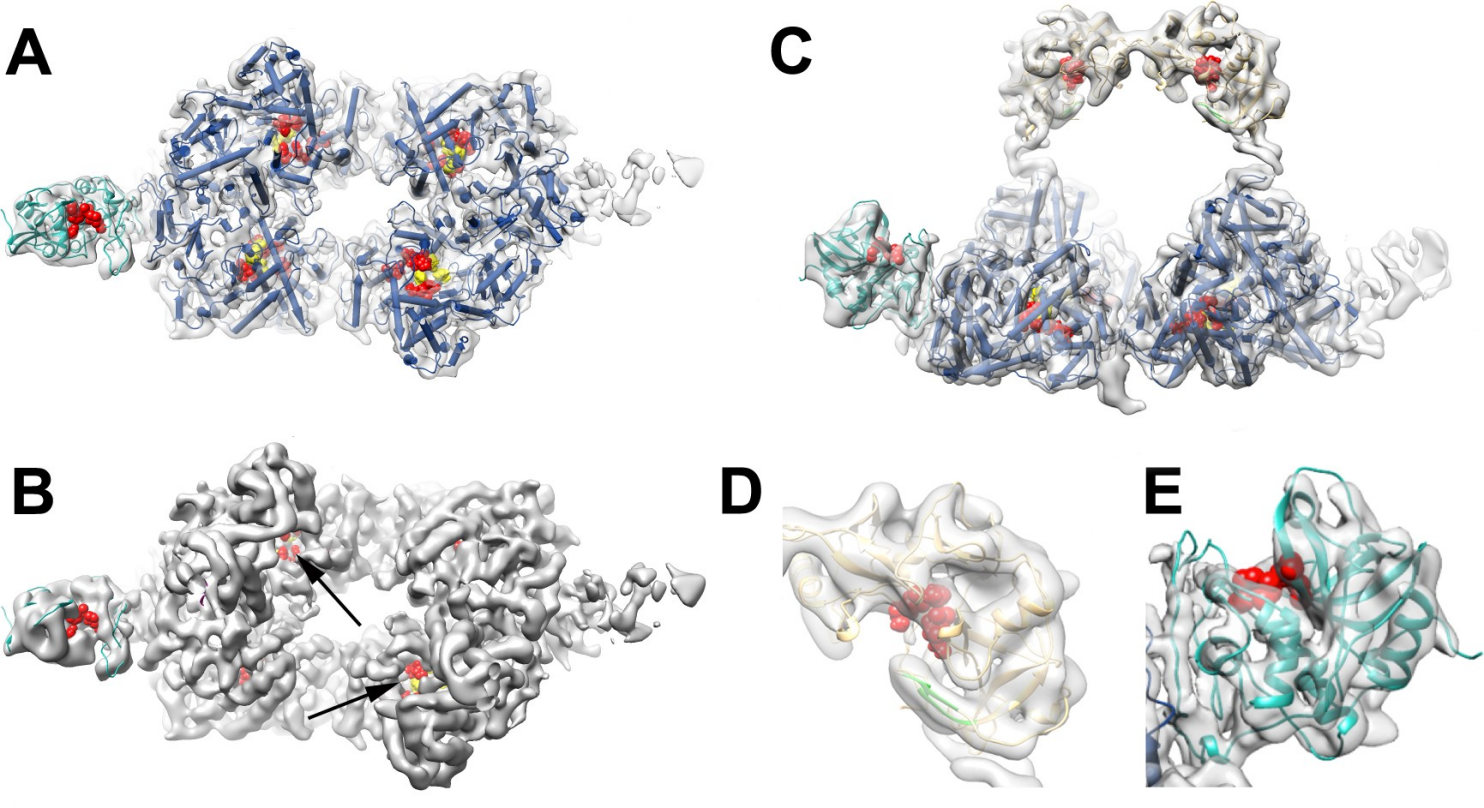
H-gal-GP is a multi-enzyme protease machine. **A** top view of the H-gal-GP complex with the arch removed with subunit active sites shown as red and yellow spheres. **B** The same view as in **A** but with only the active sites and map shown with the channels that would allow substrate to access the MEP active site and products to be transferred through the complex marked with arrows. **C** side view of the H-gal-GP complex showing the PEP1 (yellow), MEP3 (dark blue) and CP (cyan) domains with active sites highlighted by red spheres, and zinc binding region highlighted in yellow. **D** PEP1 model fitting to EM density with catalytic motif (red) and active site flap (green) highlighted. **E** CP model fitted to EM density, active site highlighted in red.

H-gal-GP is a multi-enzyme protease complex that digests the *Haemonchus* blood meal [15, 26]. The aspartyl and thiol proteases are capable of cleaving intact proteins like hemoglobin and serum albumin, major components of the *Haemonchus* blood meal, into approximately 20-mer peptides. These 20-mer peptides are the ideal substrate size for the MEPs, making for their highly efficient degradation. For example, the aspartyl proteases, located at the top of the arch (Figs 2 and 3), are likely to first cleave hemoglobin into peptides, with the metallo and cysteine proteases completing the degradation. The proposed active sites of PEP1 protrude into the central cavity of the complex (Fig 3) whose dimensions are large enough to accommodate hemoglobin (Hb) or albumin (S3 Fig). Studies on human hookworm have shown that an aspartyl protease digests intact Hb into fragments that are then digested further by a CP and MEP [27]. The proximity of the different enzymes in a close and defined organisation would allow digestion products to be passed on sequentially and rapidly, making for highly efficient degradation. The channels through the centre of the base would allow for fragments of Hb to enter the active sites of the MEPs and exit through the bottom of the base. Analysis of the predicted pepsin cleavage sites (pH>2) in ovine beta hemoglobin (Accession No. P02075) using the Peptide Cutter program (https://web.expasy.org/peptide_cutter/) [28] indicate that the largest peptide fragment generated by a typical aspartyl protease would be 21 amino acids long. This is the optimum size for further processing by members of the M13 neutral metalloendopeptidase family, as classified on the MEROPS peptidase database (https://www.ebi.ac.uk/merops/) [29].

**Fig 4:**
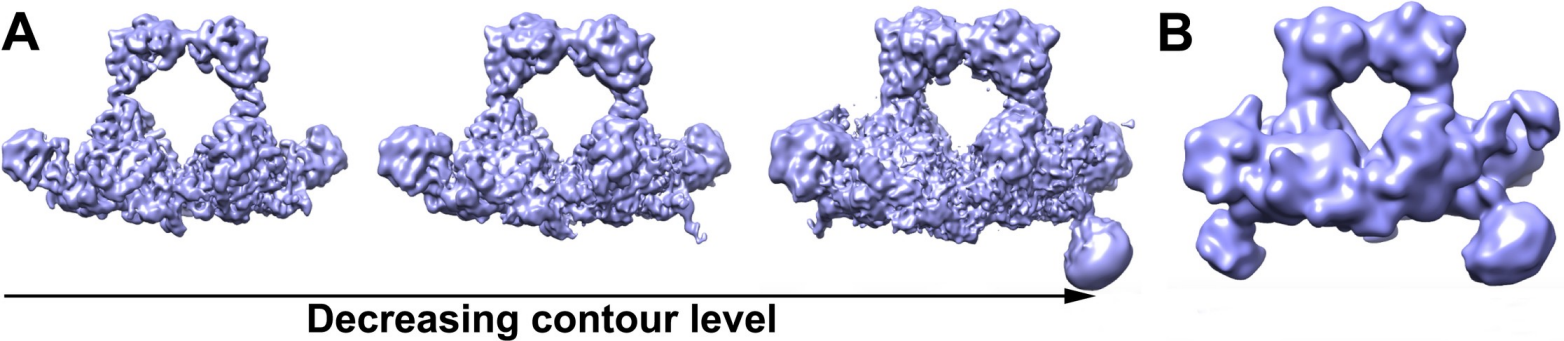
Protruding densities suggest membrane anchor position. Two-winged H-gal-GP EM density map shown at **A** varying contour levels (From left to right, UCSF Chimera Volume Viewer Level 0.07, 0.04 and 0.018, respectively). As contour levels are decreased a protrusion of density from the base is observed. At the lowest contour levels shown, this protrusion appears as a smooth ball of density, suggesting the presence of a detergent micelle. **B** H-gal-GP EM density map low-pass filtered to 15 Å resolution.

H-gal-GP is extracted directly from membranes and MEPs 1 and 3 contain elements typical of Type II integral membrane proteins, suggesting that the H-gal-GP base is embedded in the worm intestinal cell brush border membrane, but proteomic analysis shows that their N-terminal transmembrane regions are cleaved from the mature H-gal-GP complex [17]. This cleavage is supported by the EM density map which does not show any density around the N-terminus of the docked MEP3. Given a lack of detergent micelle in the reconstruction or complementary hydrophobic patch, which would accommodate the lipid bilayer, the complex is likely to be membrane-associated. Logically, if the base is associated with the membrane and associated transporters this would allow for digestion products to be efficiently transported across the membrane. Moreover, contouring the map to a lower threshold or low-pass filtering shows additional density in H-gal-GP that protrude from the base and could act as the membrane anchor. This would explain why H-gal-GP is associated with the membrane fraction and is extracted only in the presence of detergent (Fig 4).

### Flexibility and heterogeneity

**Fig 5:**
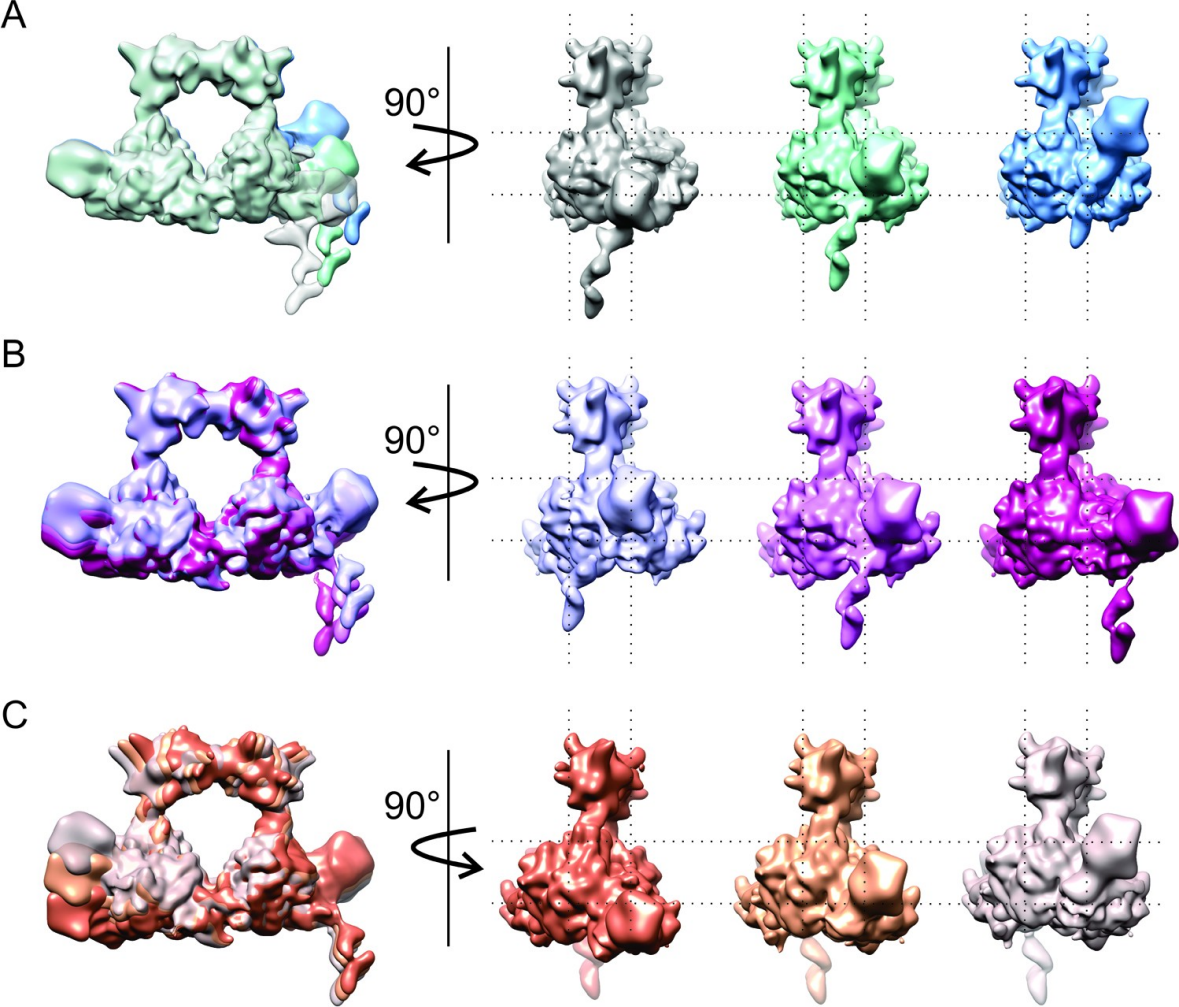
H-gal-GP is flexible. The three main ways in which H-gal-GP appears to move, as determined by principal component analysis of multi-body refinement, are illustrated in each row **A**-**C**.

2D and subsequent 3D classification of H-gal-GP particles revealed a significant amount of heterogeneity in the dataset. As well as the one-winged (4.5 Å map) and two-winged (6.2 Å map) H-gal-GP shown above, 3D classification resulted in a class containing density within the central cavity, consistent with substrate size, and a class missing the archway (representing 11% and 9% of the final particle stack respectively, S4 Fig). As well as the compositional heterogeneity described above, the reduction in resolution in the wings suggested that there was also conformational heterogeneity in H-gal-GP. To explore molecular motions, multi-body refinement was implemented, which defines different parts of the map as rigid bodies that can move independently from each other with principal component analysis defining the motions that explain the largest motion [30]. After splitting the complex into six-bodies, approximately 18% of the variance is explained by the first three principal components of variance, indicative of large conformational heterogeneity. Maps of reconstructed body densities positioned along these principal components show they correspond to significant motion in the wings and some twisting and side-ward motion in the archway (Fig 5 and S2 Movie). The role of this large motion is not known and may be exaggerated by the complex no longer residing in the native membrane environment. However, we could speculate that this motion may aid in initial substrate catching and efficient transfer of products through the complex and through the cell membrane.

### The architecture of H-gal-GP is preserved in similar complexes in other roundworms

**Fig 6:**
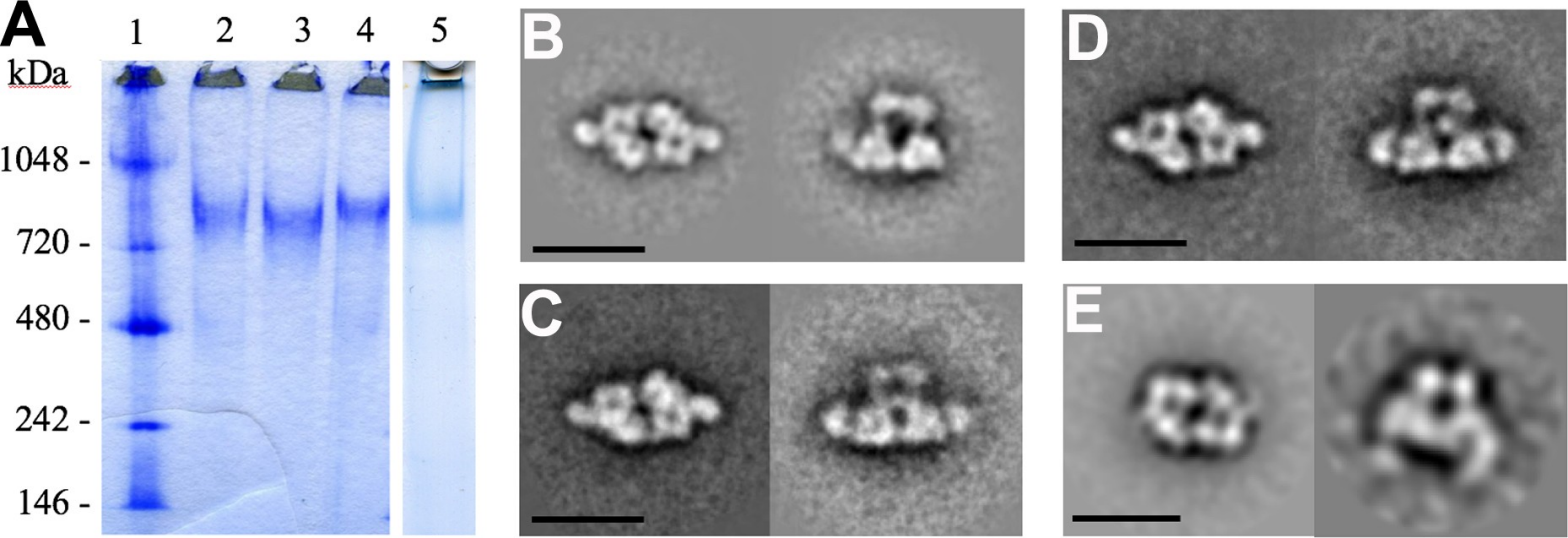
The gal-GP complex is conserved across roundworms. Complexes with similar size and architecture to H-gal-GP are found in other Strongylida roundworm parasites. **A** Blue native PAGE gel of *H*. *contortus* H-gal-GP (Lane 2), Oo-gal-GP (Lane 3), Tc-gal-GP (Lane 4) and Ac-gal-GP (Lane 5). Lane 1- molecular weight standards. End views and side views of independently refined class sums from negative-stain EM data of **B** H-gal-GP (from *H*. *contortus*), **C** Oo-gal-GP (from *O*. *ostertagi*), **D** Tc-gal-GP (from *T*. *circumcincta*) and **E** Ac-gal-GP (from *A*. *ceylanicum*) respectively.

To investigate if the architecture seen for H-gal-GP is conserved across roundworms, similar preparations were made from other nematode parasites (taxonomic Order Strongylida), Tc-gal-GP from *T*. *circumcincta*, Oo-gal-GP from *O*. *ostertagi*, and Ac-gal-GP from *A*. *ceylanicum*. Blue native gels of these revealed similar size complexes (Fig 6A), which were then examined by negative stain microscopy. H-gal-GP stain image averages were, as expected, very similar to those from cryo-EM, indicating that staining did not result in artifacts at this resolution, though preferred orientations on the carbon support were noted and the end view seen in Fig 1 (left column) was rarely observed. Image averages from Tc-gal-GP, Oo-gal-GP, and Ac-gal-GP were generated by reference-free methods in IMAGIC-5 or RELION 3.0 and all showed complexes with a similar overall architecture to H-gal-GP (Fig 6). The basic architecture is clearly conserved between the preparations from different species, consistent with high sequence similarity, which suggests similar digestive pathways in all three parasites, despite *O*. *ostertagi* and *T*. *circumcincta* not being blood feeders. Liquid-chromatography tandem mass spectrometry (LC/MS/MS) analysis of the Ac-gal-GP complex identified several families of protease directly analogous with those identified as being part of the H-gal-GP complex (S5 Table). Although the broad architecture is similar for the complexes the negative stain approach does not permit a detailed view of the different complexes with more subtle differences likely to occur between species variants. Within Ac-gal the wing densities appear to curve down and are more diffuse than those seen in H-gal-GP and other related complexes. Further studies will be needed to assess the conservation of structural features that could permit a universal vaccine approach.

## Discussion

This is the first description of a novel architecture protease complex conserved across roundworm parasites (taxonomic Order Strongylida) that is essential for nutrient uptake and a major component of the vaccine Barbervax. The arrangement of this large protease complex provides new clues into how these parasites can digest a blood meal using proteolytic enzymes embedded in the intestinal cell. Housing the proteolytic enzymes in one large complex can be a more efficient process and would retain these enzymes for extended re-use, rather than excreting them. The architecture accommodates a sequential digestion pathway, facilitating positioning of products from initial digestion for subsequent digestion by the next enzyme in the pathway. The size and shape of hemoglobin (< 70 Å^3^) and albumin (≈ 110 Å by 80 Å by 80 Å) are consistent with each entering (or partially entering) through the central chamber and we speculate that protease digestion occurs in the chamber and that resulting fragments of protein enter the cell via a narrower aperture in the base of the complex. Pepsinogen domains are located at the top of the complex and they process albumin into ~20aa peptides, the ideal substrate length for the downstream MEPs. The architecture also limits self-proteolysis and the membrane-anchor ensures the complex or the substrates being digested are not lost by excretion. The gut membrane has a significant matrix layer of ~50 Å which is consistent with the H-gal-GP complex residing on the membrane surface and not being embedded, leaving the central cavity to be exposed above the matrix layer. The anchoring of the complex in the membrane is also important to avoid host detection, since release of digestive proteins by *Haemonchus* into the hosts’ blood stream would allow for an immune response to be generated against the parasite. Therefore, the parasites may have evolved a means of efficient hemoglobin and albumin digestion that keeps the required enzymes hidden from the host.

The economic importance of H-gal-GP is highlighted by the excellent protection it gives when used as a vaccine [17]. Antibodies binding to conformational epitopes near the main cavity entrance would interfere with substrate access (S3 Fig) and indeed anti-sera from sheep immunized with H-gal-GP inhibit hemoglobin and albumin digestion *in vitro* [15]. Such conformational epitopes would exist only on the native complex, explaining the much lower protection after immunization with individual subunits [17, 19]. Because the complex is located on the microvillar surface of the parasite intestine, it is not recognized by the host during infection and is therefore a so-called ‘hidden’ antigen. Since, there has been no selection pressure for the parasite to cope with an immune response directed towards H-gal-GP, induced immunity is artificial, which explains why it is more effective than the natural immunity acquired by sheep following infection with *Haemonchus* [31].

The H-gal-GP complex is a proven vaccine target in *Haemonchus* and the presence of complexes of similar architecture in other economically and medically important parasites suggests a common ancestry and the potential for development of a unified hidden antigen vaccine. Moreover, it provides the first structural evidence for how these parasitic worms can efficiently tether the enzymes required for host meal degradation in an efficient manner without triggering a host immune response. Importantly, not all of these roundworm parasites are blood-feeding leading to the assumption that this large protease complex is not just capable of digesting and transporting a blood meal but can be adapted for a variety of substrates. Therefore, this complex has evolved to turn over a range of substrates in a variety of Strongylida and may represent a common and previously unknown mechanism for nutrient uptake.

## Materials and methods

### Preparation of H-gal-GP, H-sialgal-GP, Tc-gal-GP Oo-gal-GP and Ac-gal-GP

Complexes were purified using peanut and jacalin lectin-agarose as previously described [18, 21, 32] and stored at -20°C.

### Polyacrylamide gel electrophoresis

Blue Native gel electrophoresis was conducted as described in Smith *et al*. 1999 [16]. For Non-reducing SDS-PAGE proteins were resolved under non-reducing conditions on NuPage 4–12% gradient gels (Life Technologies), according to the manufacturer’s instructions. Native gels were subsequently stained with Coomassie blue R250.

### LC-ESI-MS/MS and database searching for Ac-gal-GP

Proteins (~7.5 μg) comprising the *A*. *ceylanicum* complex were resolved by non-reducing 1D SDS-PAGE and visualised with Coomassie Blue R250. Individual sample lanes were excised vertically from 1D SDS-PAGE gels and the resulting gel strips were cut horizontally into 25 equal gel slices 2.5 mm deep. Gel slices were then processed using standard in-gel de-staining, reduction, alkylation and trypsinolysis procedures [33]. Digests were transferred to HPLC sample vials and stored briefly at +4°C until required for liquid chromatography-electrospray ionisation-tandem mass spectrometry (LC-ESI-MS/MS) analysis. Liquid chromatography was performed using an Ultimate 3000 nano-HPLC system (Dionex) and in-line MS analysis with a 3-D high capacity ion trap tandem mass spectrometer (amaZon-ETD, Bruker Daltonics). Parameters for tandem MS analysis were based on those described previously [34]. Deconvoluted MS/MS data in .mgf (Mascot Generic Format) was imported into ProteinScape V3.1 (Bruker Daltonics) proteomics data analysis software for downstream database mining. Databases including a cognate partial *H*. *contortus* genomic sequence and the NCBInr public database (using *eukaryota* as a taxonomical search parameter) were searched utilising the Mascot V2.3 (Matrix Science) search algorithm. Mascot search parameters were set in accordance with published guidelines [35]. Molecular weight search scores attained for individual protein identifications were inspected manually and considered significant only if a) two peptides were matched for each protein, and b) each matched peptide contained an unbroken “*b*” or “*y*” ion series represented by of a minimum of four contiguous amino acid residues.

### LC-ESI-MS/MS and database searching for H-gal-GP/H-sialgal-GP

Samples (1 mg/mL) were resolved by SDS-PAGE and visualised with InstantBlue stain (Expedeon). Protein bands of interest were excised and processed as for Ac-gal-GP. Protein digests were analysed by LC-ESI-MS/MS on a NanoAcquity UHPLC -Xevo System (Waters, Manchester, UK) by the Mass Spectrometry Facility, Faculty of Biological Sciences, University of Leeds, UK. MS/MS spectra were interrogated in PEAKs and database searched against the SWISS-PROT *H*. *contortus* database. Peptide-spectrum matches were considered if they had a false discovery rate < 1% and positive protein identifications were made if two unique peptides per protein were identified.

### Negative stain electron microscopy

Negative staining used 3 μl of solution (~50 μg/ml) on carbon foil grids UV irradiated for 45 minutes immediately before use, followed by staining with 1% ^w^/_v_ uranyl acetate [36]. Grids were examined on a Technai T12 microscope operating at 120 kV and fitted with a LaB6 filament. Images were recorded at 30,000 x magnification on a 2k x 2k Gatan Ultrascan camera resulting in 4.4Å per pixel. Oo-gal-GP and Tc-gal-GP were examined on a JEOL 1200 microscope fitted with a LaB6 filament and operating at 80 kV. Images were recorded at 40,000x magnification on Kodak SO 163 film and processed in D19 developer. Films were digitised using a NIKON Coolscan at a pixel size of 2.5 Å. Target defocus was -0.7 to -1μm. Ac-gal-GP was examined on a Technai F20 microscope operating at 200 kV and fitted with a Ceta camera. Images were recorded at 30,000 x magnification at a pixel size of 3.6 Å. Data were picked using Boxer (Oo-gal-GP and Tc-gal-GP) and EMAN2 [37]^36^ (H-gal-GP, Ac-gal-GP). Images showing degradation, large defocus, or drift were removed. There were 8,221 H-gal-GP, 10,932 Oo-gal-GP, 10,043 Tc-gal-GP and 4,955 Ac-gal-GP particles. Stacks were subjected to reference-free alignment and refinement iterations in IMAGIC-5 [38] or RELION [39].

### Cryo-electron microscopy

Grids were prepared with a Vitrobot Mark IV, applying 3 μl of solution (1 mg/ml) to Quantifoil R2/1 carbon Cu 300 mesh grids (Agar Scientific, UK) glow discharged for 60 seconds prior to use (Cressington 208, Cressington Scientific Instruments, UK). Grids were blotted with Whatman no. 1 filter paper (Agar Scientific, UK) for 6 seconds at force 6, 8°C and 100% humidity, and flash-frozen in liquid ethane. Data were recorded on a FEI Titan Krios (Astbury Biostructure Laboratory, University of Leeds) operating at 300 kV with a FEI Falcon III direct electron detector. Micrographs were recorded with the EPU automated acquisition software at 75,000 x magnification, giving a final object sampling of 1.065 Å/pixel, with a total electron dose of 65 e /Å^2^, and a target defocus of 1.2–3.2 μm [40]^3^.

### Cryo-EM image processing

A summary of image processing is shown in S1 Table. Image processing was carried out using the RELION 3.0 pipeline [39]. Drift-corrected averages of each movie were created using RELION’s implementation of MotionCor2 [41] and the contrast transfer function of each determined using gCTF [42]. Approximately 1400 particles were manually picked and classified using reference-free 2D classification [43]. The resulting 2D class average views were used as templates for automated particle picking. Particles were classified using several rounds of both reference-free 2D classification and 3D classification. An initial model for 3D classification was produced from a subset of the data in RELION and filtered to 60 Å resolution for the starting model. After each round, the best classes/class was taken to the next step of classification. Post-processing was employed to appropriately mask the model, estimate and correct for the B-factor of the maps [44]. Final resolutions of H-gal-GP one-wing, two-wing and H-sialgal-GP reconstructions were determined using the ‘gold standard’ Fourier shell correlation (FSC = 0.143) criterion. Local resolution was estimated using the local resolution feature [45] in RELION. To explore molecular motions, multi-body refinement was implemented [30].

### Subunit modelling and fitting

Models for each of the protein subunits identified to be present in the H-gal-GP/H-sialgal-GP complexes by LC-ESI-MS/MS analysis were made with Phyre2, a webserver for protein modelling which uses remote homology detection methods to build 3D models [23]. The resulting 3D models were positioned into the H-gal-GP density map by use of rigid body fitting in UCSF Chimera [24]. Once docked, the MEP3 dimer and PEP1 models were refined using the molecular dynamics flexible fitting (MDFF) method [25] implemented in VMD [46]. These models were also subject to iterative cycles of refinement and model building in PHENIX and Coot, respectively [47,48]. The final statistics for the models are shown in S6 Table. Figures were generated in UCSF Chimera [24] and PyMOL [49].

## Supporting information

S1 Fig Cryo-EM data collection and processing. **A** Representative cryo-EM micrograph of H-gal-GP demonstrating a good distribution of particle and a number of different views within the ice. **B** representative classes of H-gal-GP generated in RELION that were selected for 3D refinement (scale bar represents 20nm). Fourier Shell Correlation (FSC) curves of **C** H-gal-GP two-winged and **D** H-gal-GP one-winged post-processed maps and **E** FSC curve of H-sialgal-GP post-processed map. Resolution reported to 0.143 criterion. **F** Overlay of EM density maps for H-gal-GP two-wing (yellow) and H-sialgal-GP (grey).
(TIF)

S2 Fig Subunit identification and modelling A SDS-PAGE gel of H-gal-GP and H-sialgal-GP performed under reducing conditions with lanes donated M-marker, S-H-sialgal-GP, G-H-gal-Gp. Bands extracted and analysed by LC-ESI-MS/MS are indicated by letters a-e. Overlay of models calculated with Phyre2 for **B** PEPs, **C** MEPs and the result of MDFF fitting of **D** MEP3 into the H-gal-GP EM map.
(TIF)

S3 Fig Comparison of substrate and antibody to H-gal-GP. EM density map of H-gal-GP showing models of PEP1 (purple), MEP3 (dark blue) and CP (cyan) docked into the map with ovine hemoglobin (PDB ID: 2qu0) positioned to the left and an intact antibody positioned to the right (PDB ID: 1igt filtered to 10 Å resolution) for size comparison. This highlights the complementary size of the hemoglobin substrate to the central cavity and the ability of the antibody to occlude the binding site.
(TIF)

S4 Fig EM maps for H-gal-GP produced by 3D classification show heterogeneity. Four maps are shown representing the top four classes from a classification in which the dataset were categorized into eight classes. The two-winged and one-winged H-gal-GP maps are observed as well as a map containing density within the cavity and a map lacking the archway.
(TIF)

S1 Table Cryo-EM data collection and processing. Processing statistics for the single particle cryo-EM datasets of H-gal-GP and H-sialgal-GP.
(DOCX)

S2 Table Protein identifications from LC-ESI-MS/MS analysis of H-gal-GP/H-sialgal-GP. Identification of the different H-gal-GP and H-sialgal-GP subunits using mass spectrometry.
(DOCX)

S3 Table Subunit modelling with Phyre2. Table to show the modelling statistics for the different homology models used for the study in addition to the templates used.
(DOCX)

S4 Table Molecular masses of H-gal-GP subunits and proposed H-gal-GP complexes. (DOCX)

S5 Table Protein identifications from LC-ESI-MS/MS analysis of *A*. *ceylanicum* Triton X-100 membrane extract purified by affinity chromatography with peanut lectin. (DOCX)

S6 Table Model statistics for the PHENIX refined H-gal-GP complex. (DOCX)

S1 Movie EM density of one-winged H-gal-GP colored by local resolution (as in Fig 2A) with opaque overlay of H-gal-GP two-wing density, 360° rotation in x and y. (WMV)

S2 Movie Illustration of the top three principal components accounting for motion in H-gal-GP. (WMV)
