# Supplementary figures and images for: Structure of the protective nematode protease complex H-gal-GP and its conservation across roundworm parasites
Source: PLoS Pathog. 2020 Apr 9;16(4):e1008465. doi: 10.1371/journal.ppat.1008465 (PMC7173941; doi:10.1371/journal.ppat.1008465)

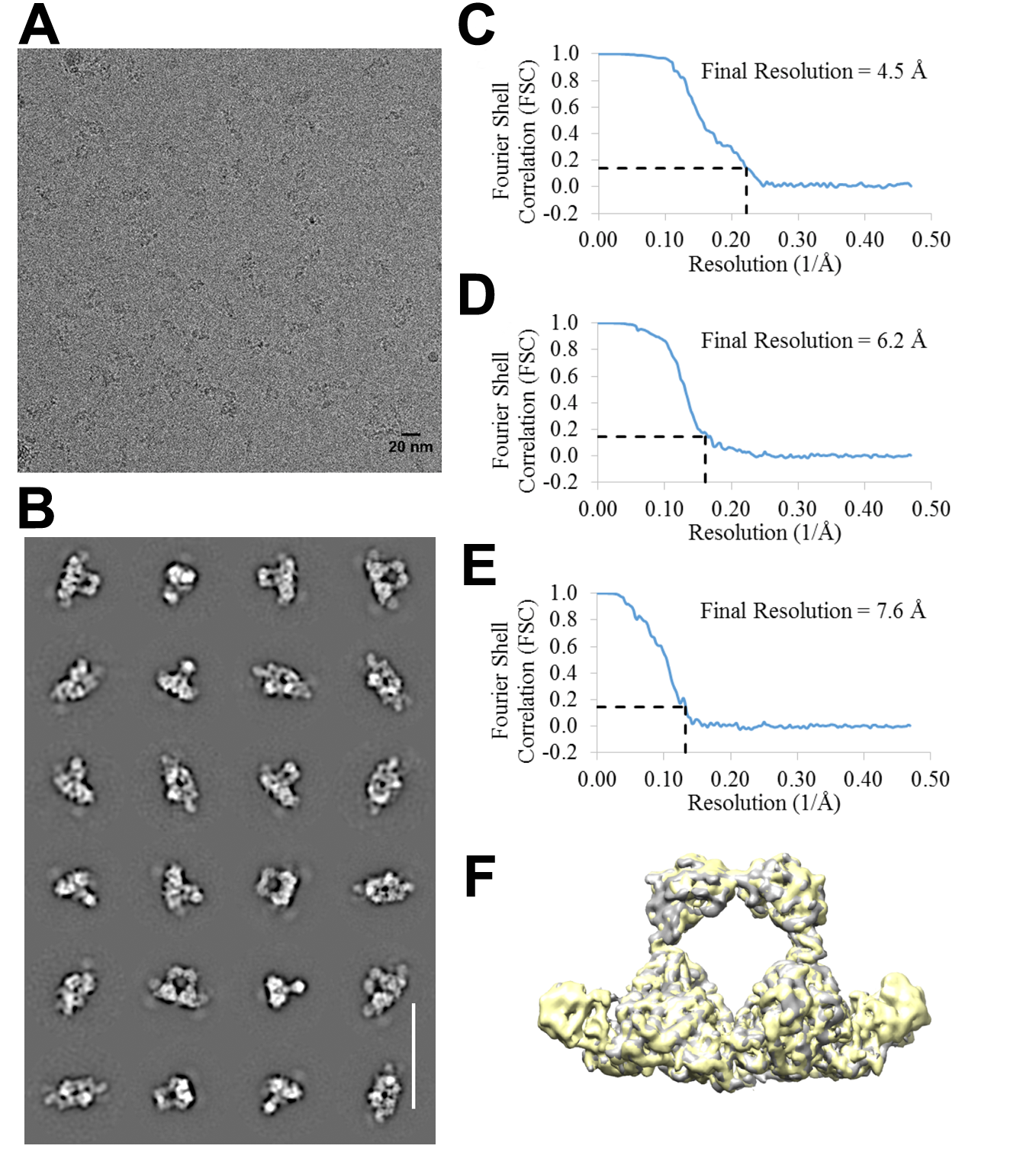

Supplement: S1 Fig — A Representative cryo-EM micrograph of H-gal-GP demonstrating a good distribution of particle and a number of different views within the ice. B representative classes of H-gal-GP generated in RELION that were selected for 3D refinement (scale bar represents 20nm). Fourier Shell Correlation (FSC) curves of C H-gal-GP two-winged and D H-gal-GP one-winged post-processed maps and E FSC curve of H-sialgal-GP post-processed map. Resolution reported to 0.143 criterion. F Overlay of EM density maps for H-gal-GP two-wing (yellow) and H-sialgal-GP (grey). (TIF) [file ppat.1008465.s001.tif]

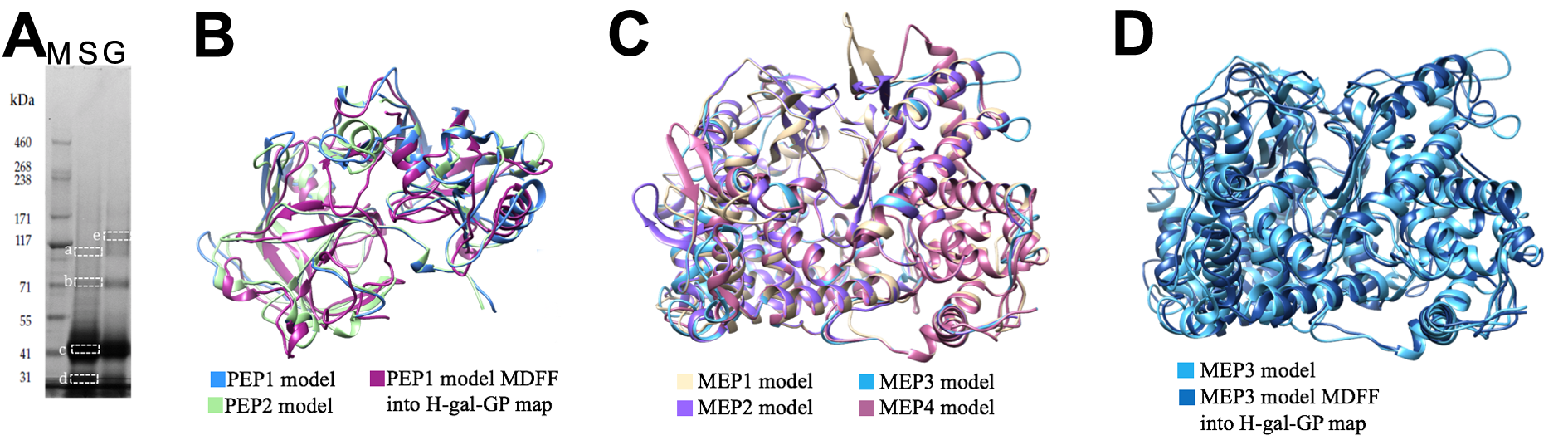

Supplement: S2 Fig — Bands extracted and analysed by LC-ESI-MS/MS are indicated by letters a-e. Overlay of models calculated with Phyre2 for B PEPs, C MEPs and the result of MDFF fitting of D MEP3 into the H-gal-GP EM map. (TIF) [file ppat.1008465.s002.tif]

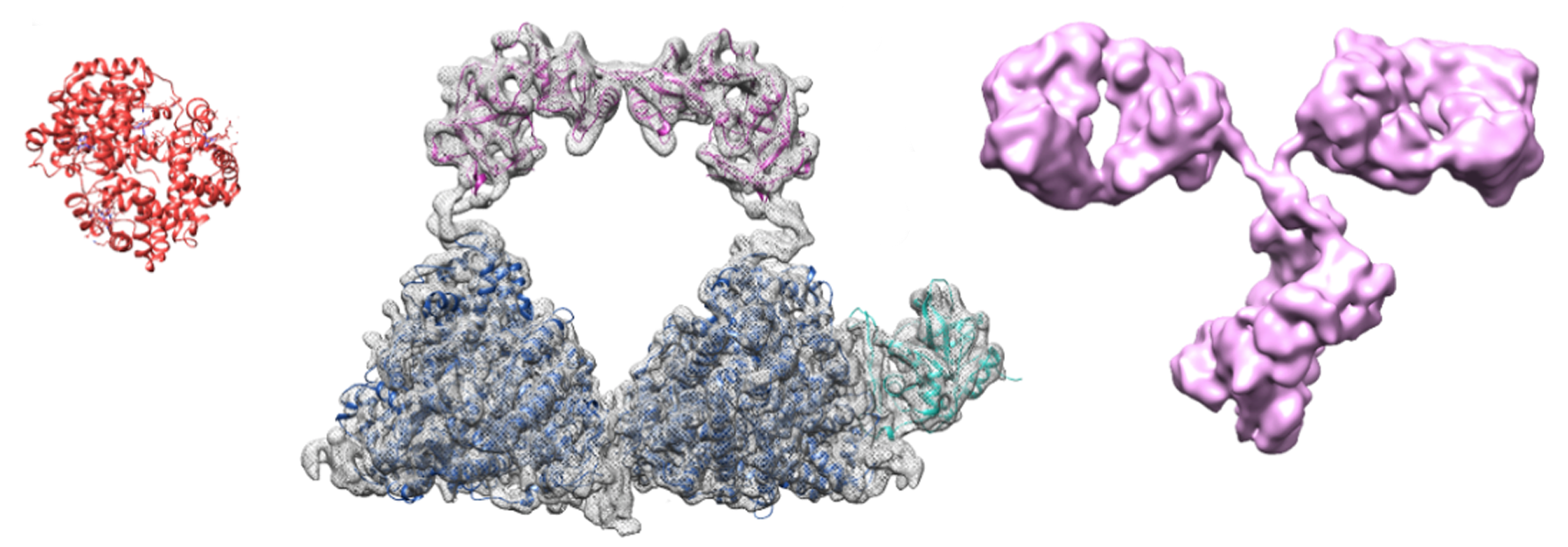

Supplement: S3 Fig — EM density map of H-gal-GP showing models of PEP1 (purple), MEP3 (dark blue) and CP (cyan) docked into the map with ovine hemoglobin (PDB ID: 2qu0) positioned to the left and an intact antibody positioned to the right (PDB ID: 1igt filtered to 10 Å resolution) for size comparison. This highlights the complementary size of the hemoglobin substrate to the central cavity and the ability of the antibody to occlude the binding site. (TIF) [file ppat.1008465.s003.tif]

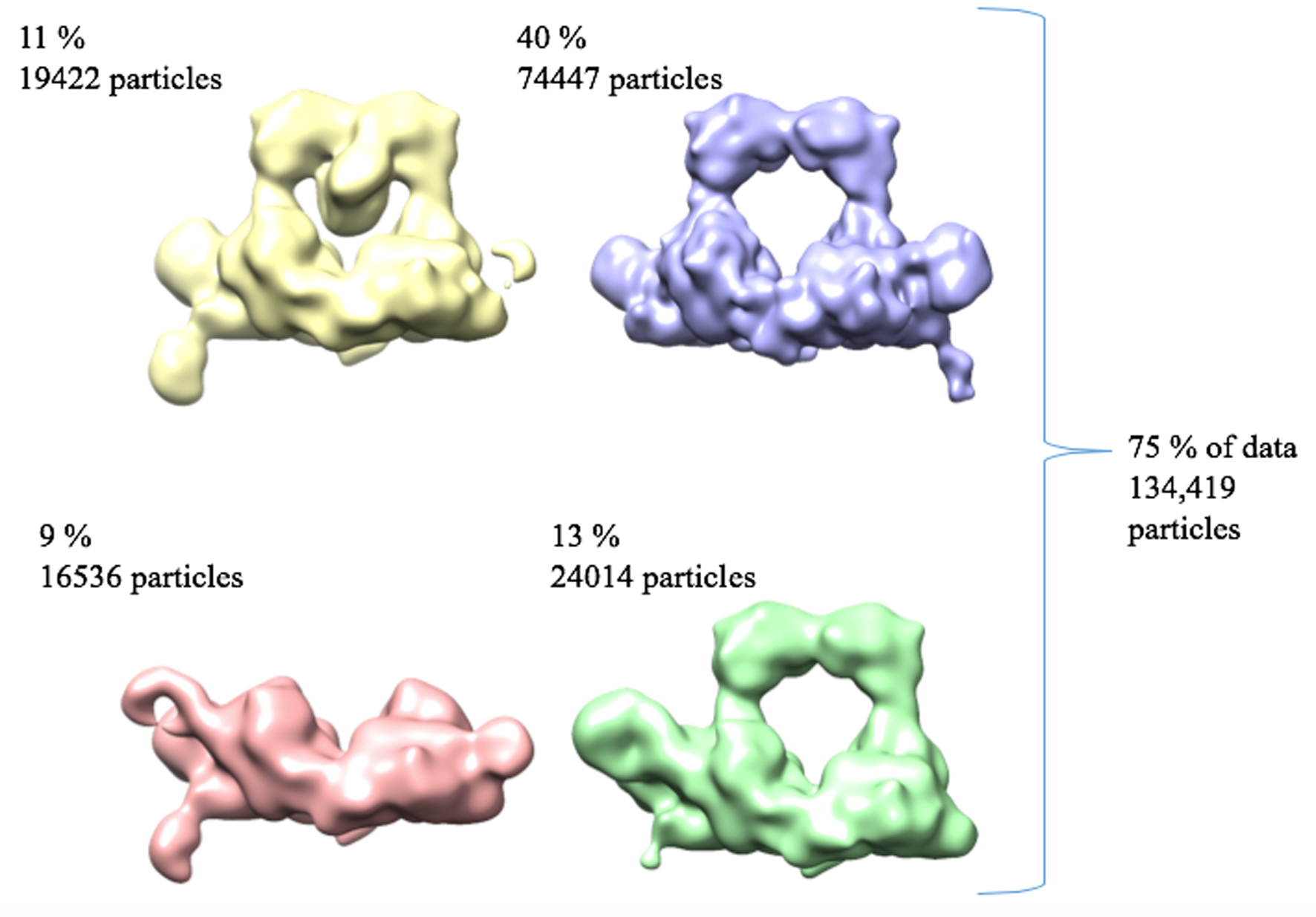

Supplement: S4 Fig — Four maps are shown representing the top four classes from a classification in which the dataset were categorized into eight classes. The two-winged and one-winged H-gal-GP maps are observed as well as a map containing density within the cavity and a map lacking the archway. (TIF) [file ppat.1008465.s004.tif]
