# Supplementary material for: Structure of the protective nematode protease complex H-gal-GP and its conservation across roundworm parasites
Source: PLoS Pathog. 2020 Apr 9;16(4):e1008465. doi: 10.1371/journal.ppat.1008465 (PMC7173941; doi:10.1371/journal.ppat.1008465)
Supplement: S1 Table — Processing statistics for the single particle cryo-EM datasets of H-gal-GP and H-sialgal-GP. (DOCX) [file ppat.1008465.s005.docx]

| **Data collection/ processing parameter** | **H-gal-GP two-wing** | **H-gal-GP one-wing** | **H-sialgal-GP** |
| --- | --- | --- | --- |
| Magnification | 75,000 x | 75,000 x | 75,000 x |
| Voltage (kV) | 300 | 300 | 300 |
| Electron exposure (e-/Å2) | 62.5 | 62.5 | 62.4 |
| Defocus range (µm) | -1.2 to -3.2 | -1.2 to -3.2 | -1.2 to -3.2 |
| Pixel size | 1.065 | 1.065 | 1.065 |
| Initial particle number | 180,100 | 180,100 | 202,778 |
| Final particle number | 53,590 | 110,863 | 23,186 |
| Map resolution (Å) | 6.2 | 4.5 | 7.5 |
| FSC threshold | 0.143 | 0.143 | 0.143 |
| Map resolution range | 5.2-8.9 | 4.2-6.5 | 6.2-15.2 |
