# Supplementary material for: Structure of the protective nematode protease complex H-gal-GP and its conservation across roundworm parasites
Source: PLoS Pathog. 2020 Apr 9;16(4):e1008465. doi: 10.1371/journal.ppat.1008465 (PMC7173941; doi:10.1371/journal.ppat.1008465)
Supplement: S2 Table — Identification of the different H-gal-GP and H-sialgal-GP subunits using mass spectrometry. (DOCX) [file ppat.1008465.s006.docx]

| **Sample (gel band)†** | **Protein name** | **UniProtKB ID** | **No. of tryptic peptides identified** | **No. of unique peptides identified** | **Sequence coverage (%)** |
| --- | --- | --- | --- | --- | --- |
| H-sialgal-GP (a/b/**c**/d) | MEP1 | Q25051 | 8/8/**65**/15 | 7/6/**58**/13 | 8/8/**60**/18 |
| H-sialgal-GP (a/**b**/c/d/e) | MEP2 | O76750 | 3/**18**/13/9/3 | 3/**15**/11/9/3 | 3/**23**/19/12/3 |
| H-sialgal-GP (a/**d**) | MEP3 | O76751 | 6/**14** | 5/**12** | 6/**14** |
| H-gal-GP (**e**) | MEP4 | Q9Y1I4 | **16** | **16** | **28** |
| H-sialgal-GP (a/**c**/e) | PEP1 | Q25037 | 3/**24**/3 | 3/**23**/3 | 8/**60**/8 |
| H-sialgal-GP (**d**) | PEP2 | Q70JE2 | **15** | **15** | **36** |
| H-sialgal-GP (**d**) | CP | U6P4Z7 (GenBank: CDJ88569.1) | **14** | **14** | **27** |

**†**As described in Figure S2.
