# Supplementary material for: Structure of the protective nematode protease complex H-gal-GP and its conservation across roundworm parasites
Source: PLoS Pathog. 2020 Apr 9;16(4):e1008465. doi: 10.1371/journal.ppat.1008465 (PMC7173941; doi:10.1371/journal.ppat.1008465)
Supplement: S3 Table — Table to show the modelling statistics for the different homology models used for the study in addition to the templates used. (DOCX) [file ppat.1008465.s007.docx]

| **Protein**  **name†** | **Submitted sequence start-end * (amino acid no.)** | **Molecular mass of submitted sequence (kDa)** | **% of sequence modelled with 100 % confidence** | **Main templates for model (first five)** | **Template family** | **Model start-**  **end (amino acid no.)** |
| --- | --- | --- | --- | --- | --- | --- |
| MEP1 | 76-823 | 85.7 | 91 | d1dmta; c3dwbA; c3zukB; c4iuwA; d1bswa | Metalloproteases, neutral endopeptidase | 92-820 |
| MEP2 | 83-747 | 75.9 | 91 | As for MEP1 | As for MEP1 | 99-747 |
| MEP3 | 71-837 | 87.9 | 91 | As for MEP1 | As for MEP1 | 81-835 |
| MEP4 | 33-938 | 103.2 | 75 | As for MEP1 | As for MEP1 | 200-936 |
| PEP1 | 59-428 | 40.8 | 85 | d1miqa; c3qvcA; d1pfza; c1qdmA; d3psga | Pepsin-like proteases | 59-427 |
| PEP2 | 59-427 | 41.4 | 86 | d1miqa; c3qvcA; d1pfza; c1qdmA; d3psga | Pepsin-like proteases | 59-426 |
| CP | 86-339 | 28.4 | 99 | d3pbha; c3qvcA; d1mira; c4hwyA; d2dcca1 | cysteine proteases, papain-like; hydrolase | 86-338 |

**†** UniProtKB ID’s as in Table S2. *based on evidence for expressed protein sequence from N-terminal sequencing and pre-proenzyme predicted cleavage site for PEP1 [16].
