# Supplementary material for: Structure of the protective nematode protease complex H-gal-GP and its conservation across roundworm parasites
Source: PLoS Pathog. 2020 Apr 9;16(4):e1008465. doi: 10.1371/journal.ppat.1008465 (PMC7173941; doi:10.1371/journal.ppat.1008465)
Supplement: S4 Table — (DOCX) [file ppat.1008465.s008.docx]

| Protein Name† | Molecular Mass * (kDa) | Proposed Glycan Mass (kDa)^↑^ | Complex Mass †(kDa) |
| --- | --- | --- | --- |
| MEP1 | 86 | ≈12 | 86-98 |
| MEP2 | 76 | ≈10 | 76-86 |
| MEP 3 | 88 | ≈15-17 | 176-210 |
| MEP 4 | 103 | ≈16 + O-linked | 103-119 |
| PEP1 | 41 | ≈2 | 82-86 |
| PEP2 | 41 | ≈2 | 83-87 |
| Cysteine protease | 28 | 0 | 56 |
| H-gal-GP complexes |  |  | 493-570 |

† UniProtKB ID’s as in Table S2. * based on evidence for expressed protein sequence from N-terminal sequencing and pre-proenzyme predicted cleavage site for PEP1 [16]. † Assuming two copies of MEP3, as gel analysis suggests [16,18] and either a MEP1/4 or MEP1/2 dimer, two copies of either PEP1 or PEP2 and two CPs as suggested by the H-gal-GP cryo-EM map. ^↑^.
