# Supplementary material for: Structure of the protective nematode protease complex H-gal-GP and its conservation across roundworm parasites
Source: PLoS Pathog. 2020 Apr 9;16(4):e1008465. doi: 10.1371/journal.ppat.1008465 (PMC7173941; doi:10.1371/journal.ppat.1008465)
Supplement: S5 Table — (DOCX) [file ppat.1008465.s009.docx]

| **H-gal-GP component** | ***A. ceylanicum* protein accession No.** |
| --- | --- |
| MEP1 | EPB70588.1 |
| MEP 3 | EPB70589.1 |
| MEP 4 | EPB70590.1 |
| PEP1 | EPB79890.1 |
| PEP2 | EPB71712.1 |
| Cysteine protease | EPB68353.1, EPB71584.1, EPB71593.1,  EPB72620.1, EPB72621.1, EPB72622.1 |
