# Supplementary material for: Structure of the protective nematode protease complex H-gal-GP and its conservation across roundworm parasites
Source: PLoS Pathog. 2020 Apr 9;16(4):e1008465. doi: 10.1371/journal.ppat.1008465 (PMC7173941; doi:10.1371/journal.ppat.1008465)
Supplement: S6 Table — (DOCX) [file ppat.1008465.s010.docx]

| **Parameter** | ***Value*** |
| --- | --- |
| Poor rotamers | 0 |
| Favoured rotamers | 1994 (96.8%) |
| Ramachandran outliers | 29 (0.98%) |
| MolProbidity Score | 2.98 |
| Bad bonds | 13/23286 (0.06%) |
| Bad angles | 40/31501 (0.13%) |
| Cis Prolines | 0 |
